# Stroop interference in children with developmental dyslexia: An event-related potentials study

**DOI:** 10.1097/MD.0000000000026464

**Published:** 2021-06-25

**Authors:** Chengwei Shen, Qi Jiang, Yan Luo, Ji Long, Xiujuan Tai, Shuqing Liu

**Affiliations:** a Department of Psychology, School of Medical Humanitarians; b Guiyang Maternal and Child Health Care Hospital; c School of Public Health, Guizhou Medical University, Guiyang, China.

**Keywords:** developmental dyslexia, event-related potentials, executive attention, inhibition, Stroop

## Abstract

Previous studies have identified inhibitory deficits in dyslexic children, but we have little understanding of their neural mechanisms, especially for Chinese children with developmental dyslexia.

We used a double-blind controlled trial to study the electroencephalogram responses of dyslexic and non-dyslexic children when performing the Stroop color-word test.

Behavioral data showed differences in response time and accuracy between the 2 groups. In the event-related potentials (ERP) results, dyslexic children displayed larger P2 and P3b on congruent trials, while non-dyslexic children displayed larger P2 and P3b on incongruent trials, the 2 groups showed opposite brain activation patterns on the Stroop test.

Dyslexic children have poor inhibitory function, and this poor inhibition may be related to their abnormal brain activation patterns.

## 1 Introduction

Developmental dyslexia (DD), which is a special learning disorder caused by the nervous system problems, is mainly manifested as difficulty in reading, decoding, and spelling words.^[1]^ The reading ability of DD children is significantly worse than that of typically developing children, even if they have the same educational opportunity.^[2]^ Study shows the cumulative incidence of DD is 5% to 12%^[3]^ and DD impairs a child's ability to read and write. Phonological awareness defect is considered to be the core defect of DD children using alphabetic language. As a non-alphabetic language, Chinese is different from alphabetic language, and Chinese DD children have different types of defects compared with English DD children.^[4]^

Because of the late-starting on researching Chinese dyslexia and using inconsistent standards, no consensus has been reached on the core defects of Chinese DD children. Previous studies have found that children with Chinese DD have defects in phonological awareness, orthographic awareness, and working memory.^[5,6]^ Recent studies have found that defects of DD arise from cognitive processing.^[7]^ Research revealed that in general cognitive ability, DD is highly correlated with working memory.^[8]^

Working memory refers to an individual's ability to temporarily retains and manipulates information while performing cognitive tasks.^[9]^ The most influential model of working memory describes it as being composed of 4 subsystems. Central executive is the core of working memory, connecting between the various subsystems, assigning cognitive resources for specific tasks, and processing the information stored in the working memory. Phonological loop is a voice information processor storing phonological information and preventing its decay by continuously refreshing it in a rehearsal loop. Visuospatial sketchpad stores visual and spatial information. It can be used, for example, for constructing and manipulating visual images and representing mental map. Episodic buffer is a multi-modal coding storage system controlled by the central execution system.

People with DD have defects in the 4 subsystems of working memory,^[10]^ and they also have defects in updating, shifting, and inhibition of the central executive system.

Inhibition is important for the central execution system, the ability to inhibit irrelevant information plays a crucial role in several aspects of cognitive development, including learning, memory, and reading comprehension. Smith-Spark et al^[11]^ found that people with DD self-reported more executive function problems in their daily life and showed significant inhibitory deficits.

Reading theory emphasizes the crucial role of behavior inhibition in the reading process,^[12]^ and poor behavior inhibition may result in poor recognition of letters and words. For example, children with DD must inhibit incorrect correspondence between orthography and pronunciation (e.g., read b as d and p as q). If this incorrect pronunciation cannot be inhibited, it may result in impaired reading ability. Lobier et al^[13]^ found that DD children have significant deficiencies in attention shift, attention distribution, and attention span. Compared with non-dyslexic children, DD children are more likely to be attracted by irrelevant information due to selective attention deficit.

Inhibition refers to the ability to focus selectively on relevant information and resolve conflicting responses while disregarding irrelevant information.^[14]^ Dysfunctional inhibition has been associated with DD. Studies using the Stroop color-word test have indicated poor Stroop test performance in DD children.^[15]^ There are 2 types of trials in the Stroop color-word test: congruent trial (e.g., RED printed in red) and incongruent trial (e.g., GREEN printed in red), and participants are asked to inhibit the dominant response (reading the word) and say the non-dominant response (name the color ink). Wu et al^[16]^ found that children with Chinese dyslexia performed worse than non-dyslexic children on the Stroop test, and their ability to inhibit irrelevant information was also worse than non-dyslexic children.

Inhibition can be reflected not only in behavior, but also in the cerebral cortex. Since inhibition is a very rapid response, only by studying the cerebral activity of DD children when they are performing inhibiting test can we understand more specifically of the inhibitory deficits of DD children.

Event-related potentials (ERP) with high time resolution can be utilized to observe brain activities at any time period. In addition, ERP technology is fairly simple and non-invasive, and ERP is suitable for children's brain function research. Therefore, we recorded the behavior and ERP responses of DD children and non-dyslexic children when performing the Stroop test.

We make the following hypotheses: the reaction time (RT) of DD children is longer than that of non-dyslexic children and the accuracy is lower than that of non-dyslexic children. We believe that due to inhibitory deficits, DD children need to invest more cognitive resources when performing the Stroop test, so the ERP components of DD children should be larger than non-dyslexic children (we found that the ERP components of the Stroop test found in previous studies are inconsistent,^[17]^ so we analyzed the ERP components obtained in the present study).

As far as we know, this is the first published ERP study on the inhibitory function of children with Chinese DD. On the one hand, the results can provide us with more information about the neural mechanisms of children with Chinese DD. On the other hand, the results can provide training guidance on improving the reading ability of children with Chinese DD.

## 2 Methods

### 2.1 Participants

Thirty eight participants were recruited from grade 3 to 5 at a primary school in Guiyang. We excluded 4 children (1 for too many artifacts, and 3 for low accuracy). Seventeen children with DD (9 boys with an average age of 11.41 ± 0.93 years) participated in the study as the experimental group, and 17 non-dyslexic children (9 boys with an average age of 11.47 ± 0.52 years) as the control group.

All children were native speakers of Chinese, with normal or corrected-to normal vision and no history of brain injury or neurological problems. All the children completed the Raven's Standard Progressive Matrices, and the children with scores >50% have been selected. Clinicians at Guiyang Maternal and Child Health Hospital in China diagnosed each child with dyslexia in the experimental group. There was no significant difference in age, sex, and intelligence quotient between the 2 groups (*P* > .05).

We used a double-blind, paired designed study. The study was approved by the local ethics committee (School of Medical Humanitarians, Guizhou Medical University), and written informed consent was obtained from the parents or guardians of the participating children.

### 2.2 Stimulus and procedure

A computerized Stroop color-word test, adapted from previous research,^[18]^ was used as the test of central executive function, especially inhibition function. Subjects were comfortably seated in a quiet room with their eyes 80 cm from the computer screen.

The color-words were displayed on a flat 14-in. monitor. The Stroop test consisted of 2 types of trials (i.e., congruent and incongruent trials). The stimuli were the Chinese words (RED), (YELLOW), (BLUE), and (GREEN), which were printed in 1 of these 4 colors on a black background, so that the stimulus features (word color and word meaning) were either congruent (the color and meaning matched) or incongruent (the word and color did not match), half of the stimuli were congruent and half incongruent. Before the experimental test, there were 10 trials of practice. In the experimental test, 240 trials of the Stroop test were divided into 4 blocks with 5-minute intervals after each 60 trials, congruent trials and incongruent trials were randomly assigned to each block. The presentation time for each stimulus was 150 ms, and they had 1900 ms to respond. Participants were asked to respond to the color of the word (red-1, yellow-2, blue-3, and green-4) before the next stimulus appeared, a 500 ms blank screen with a white cross in the middle will be displayed first, requiring the participants to concentrate on the screen. We only analyzed the RT and accuracy of the correct response trials.

### 2.3 EEG recording and preprocessing

The electroencephalogram (EEG) was recorded continuously from 40 scalp sites at a sampling rate of 1000 Hz. The electrodes were distributed according to the international 10 to 20 system with scalp resistance at each electrode was kept under 5 kΩ, and the band pass was 0.1 to 100 Hz. During the acquisition, the data were amplified with a Neuroscan SynAmps amplifier.

The recording electrodes were FP1, FP2, F7, F3, Fz, F4, F8, FT7, FC3, FCz, FC4, FC8, T3, C3, Cz, C4, T4, TP7, CP3, CPz, Cp4, TP8, T5, P3, Pz, P4, T6, O1, Oz, O2. In addition, to monitor ocular artifacts, vertical and horizontal electrooculographic potentials were recorded bipolarly. The data were re-referenced to the average of the right and left mastoid offline. The pretreatment and analysis of EEG signals were carried out in Matlab (versionR2018b; MathWorks, Inc., MA).

The EEG signals were passed through a 0.1 to 30 Hz band-pass filter. Segments of 800 ms (200 ms pre-stimulus) were extracted from the EEG, and the pre-stimulus interval was defined as the baseline. Signals exceeding ±100 μV were automatically excluded from the averages. Finally, artifact-free epoch segments were averaged for each type of Stroop trials (congruent trials and incongruent trials).

### 2.4 Data analysis

Based on previous research^[17]^ and visually inspecting the waveforms and topographic map, P2 was found to peak approximately 200 ms after the onset of stimuli and was mainly distributed in the frontal area. P3b is a positive-going amplitude peaking at 250 to 500 ms. We found the amplitude of P3b is usually highest on the scalp in parietal brain regions.

The electrodes we chose for the P2 component were Fz, FCz, Cz, and for the P3b component were CPz, Pz, Oz. The peak latencies and amplitude of P2 and P3b were examined by repeated measurement analysis of variance (ANOVA), with group as the between-subject factor (2 levels: DD and non-dyslexic), and stimulus type as the within-subject factor (2 levels: congruent and incongruent), and Electrode position as within-subject factors (3 levels for P2: Fz, FCz, and Cz and 3 levels for P3b: CPz, Pz, and Oz).

RT and accuracy were examined by repeated measurement ANOVA, with group as the between-subject factor (2 levels: DD and non-dyslexic), and stimulus type as the within-subject factor (2 levels: congruent and incongruent).

Behavioral and ERP data were analyzed by SPSS 23 (IBM Corp, Armonk, NY), and for all the results that did not meet the hypothesis of spherical test, the Greenhouse-Geisser method was used to correct the *P*-value, and *P*-value ≤.05 was considered significant.

## 3 Results

### 3.1 Behavioral results

#### 3.1.1 Reaction time

Significant main effects of group (*F* [1,32] = 6.44, *P* = .016) and stimulus type (*F* [1,32] = 96.20, *P* = .000) were found, indicating longer RT for the DD group compared with the non-dyslexic group and longer RT for the incongruent trials compared with the congruent trials for the both groups. The interaction between group and stimulus type was not significant (*F* [1,32] = 2.80, *P* = .11) (see Table 1).

**Table 1:** Summary statistical analysis of behavioral indices, mean values, and standard deviations (in brackets).

	Group	
Measures	DD group	Non-dyslexic group
Reaction time, ms		
Congruent	692.62 (98.51)	586.10 (80.82)
Incongruent	776.03 (122.76)	703.76 (120.13)
Accuracy (%)		
Congruent	78 (11)	86 (07)
Incongruent	72 (13)	81 (11)

DD = developmental dyslexia.

#### 3.1.2 Accuracy

Results indicated significant main effects of group (*F* [1,32] = 4.86, *P* = .035) and stimulus type (*F* [1,32] = 25.32, *P* = .000). The non-dyslexic group was more accurate than the DD group. The congruent trials were more accurate than the incongruent trials. No significant group × stimulus type interaction (*F* [1,32] = 0.153, *P* = .70) was obtained.

### 3.2 ERP results

#### 3.2.1 P2 amplitude and latencies

##### 3.2.1.1 Amplitude

**Figure 1:**
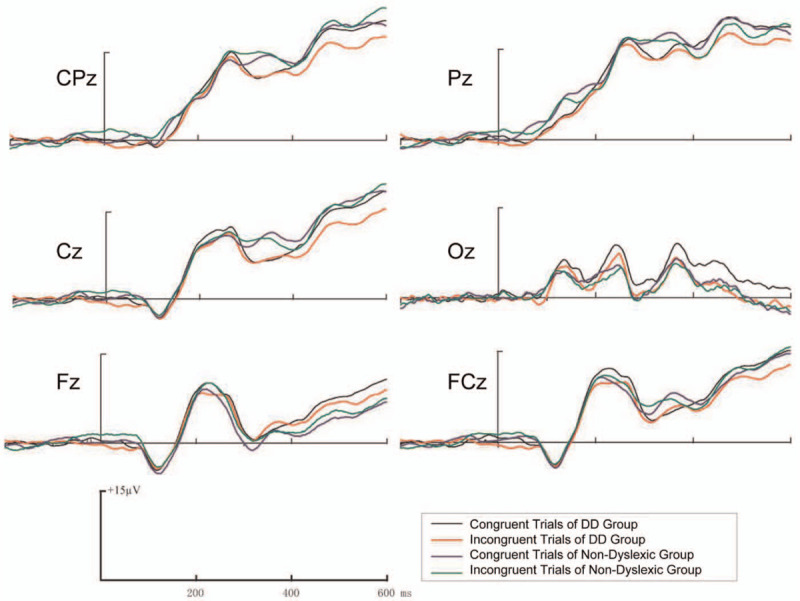
Grand average ERP waveforms, at Fz, FCz, Cz, CPz, Pz, and Oz electrode sites, elicited by congruent color words and incongruent color words in the DD group and non-dyslexic group. DD = developmental dyslexia, ERP = event-related potentials.

**Figure 2:**
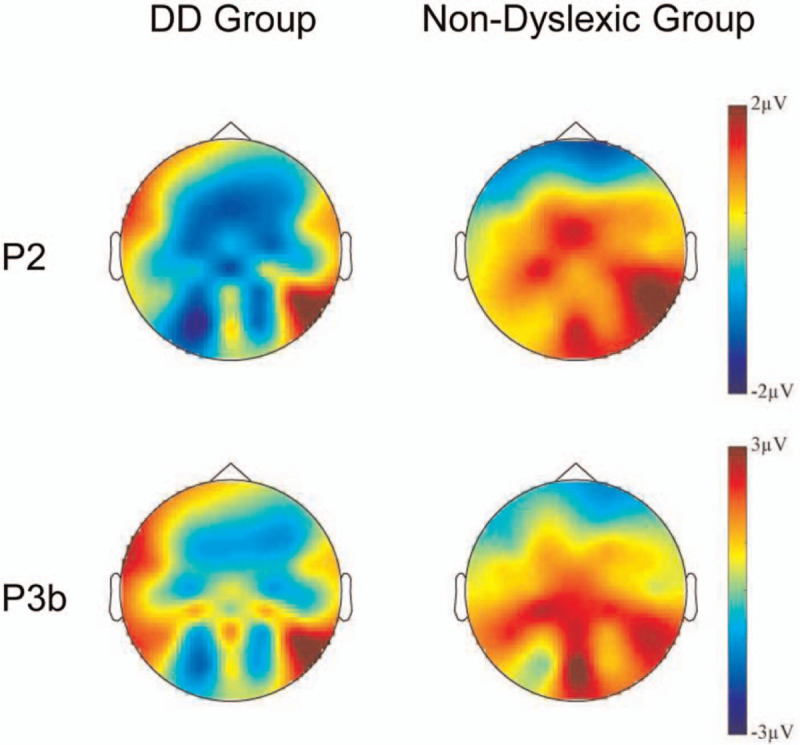
Topographical maps of voltage amplitudes for the incongruent versus congruent color word difference wave for P2 (200–300 ms) and P3b (400–500 ms), incongruent minus congruent.

**Figure 3:**
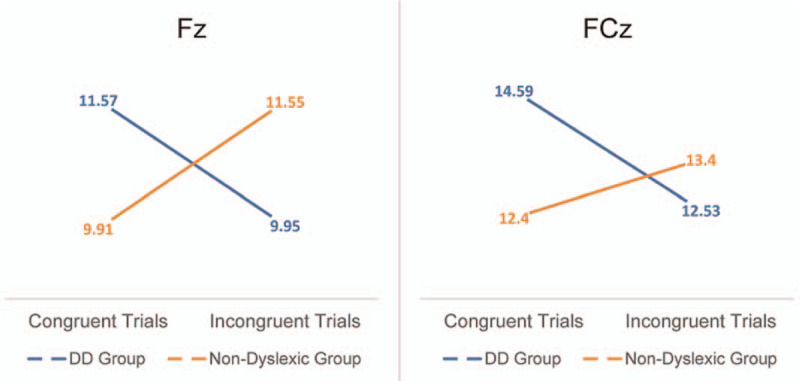
Interaction for P2 amplitudes of congruent and incongruent stimuli in DD and non-dyslexic group. DD = developmental dyslexia.

A significant main effect of electrode position (*F* [2,31] = 8.33, *P* = .001) was found. There was a significant group × stimulus type interaction (*F* [1,32] = 6.53, *P* = .016). Moreover, we conducted a repeated measures ANOVA on P2 at FCz revealed a significant group × stimulus type interaction (*F* [1,32] = 7.37, *P* = .11). This interaction stemmed from the amplitude of the congruent trials of the DD group was significantly larger than the incongruent trials (*F* [1,32] = 6.68, *P* = .015). We conducted a repeated measures ANOVA on P2 at Fz revealed a significant group × stimulus type interaction (*F* [1,32] = 9.35, *P* = .004). This interaction stemmed from the amplitude of the incongruent trials (*F* [1,32] = 4.97, *P* = .033) was significantly larger than the congruent trials of the non-dyslexic group, while the amplitude of the congruent trials (*F* [1,32] = 4.83, *P* = .03) was significantly larger than the incongruent trials of the DD group. (see Figs. 1 and 2. The results are shown in Fig. 3, see Table 2).

**Table 2:** Summary statistical analysis of P2 and P3b, mean values, and standard deviations (in brackets).

		DD group				Non-dyslexic group			
Measures		Amplitude, μV		Latency, ms		Amplitude, μV		Latency, ms	
		Congruent	Incongruent	Congruent	Incongruent	Congruent	Incongruent	Congruent	Incongruent
	Fz	11.57 (4.03)	9.95 (4.44)	283.76 (23.19)	247.94 (25.90)	9.91 (4.45)	11.55 (5.99)	229.35 (18.80)	233.35 (19.08)
P2	FCz	14.59 (3.76)	12.53 (5.06)	236.24 (27.31)	246.06 (27.72)	12.40 (4.57)	13.40 (4.25)	231.47 (24.70)	232.47 (25.64)
	Cz	15.17 (4.66)	14.04 (6.73)	249.71 (24.64)	254.76 (25.78)	13.08 (6.99)	14.42 (5.88)	247.35 (25.38)	249.71 (24.64)
	CPz	19.87 (6.38)	17.93 (9.27)	466.59 (27.76)	475.71 (29.98)	20.16 (9.34)	20.80 (4.13)	461.24 (26.32)	470.53 (29.98)
P3b	Pz	21.40 (7.35)	18.65 (9.34)	466.79 (23.31)	469.11 (20.41)	21.66 (7.89)	20.39 (7.35)	463.47 (20.47)	475.76 (27.31)
	Oz	7.83 (4.66)	3.91 (9.32)	442.47 (19.50)	443.24 (25.14)	5.00 (6.99)	4.28 (6.37)	447.76 (30.77)	451.35 (30.18)

DD = developmental dyslexia.

##### 3.2.1.2 Latency

There was a significant main effect of electrode position (*F* [2,31] = 9.45, *P* = .001), and no significant interaction. We conducted a repeated measures ANOVA on P2 at Fz and revealed significant effects of stimulus type (*F* [1,32] = 4.95, *P* = .033), with incongruent trials having significantly longer latencies than congruent trials.

#### 3.2.2 P3b amplitude and latencies

##### 3.2.2.1 Amplitude

A significant main effect of electrode position (*F* [1,32] = 96.02, *P* = .000) was found. Furthermore, we conducted a repeated measures ANOVA (group × stimulus type) on P3b at Pz. In the DD group, the amplitude was larger for congruent trials than for incongruent trials (*F* [1,32] = 4.57, *P* = .04). There was no significant difference between congruent trials and incongruent trials in the non-dyslexic group (*F* [1,32] = 0.97, *P* = .033) (see Table 2).

##### 3.2.2.2 Latency

There was a significant main effect of electrode position (*F* [2,31] = 17.603, *P* = .000). We conducted a repeated measures ANOVA on P3b at the Pz, and found a significant Group × stimulus type interaction (*F* [1,32] = 7.37, *P* = .011). This interaction stemmed from the fact that incongruent trials in the non-dyslexic group had significantly longer latencies than congruent trials (*F* [1,32] = 7.67, *P* = .009).

## 4 Discussion

On the ERP data of the present study, we found an interesting result. In terms of P2 and P3b components, we noted differences between the DD group and the non-dyslexic group of the ERP amplitude in response to congruent trials and incongruent trials. Overall, the ERP amplitude of the non-dyslexic group was larger on incongruent trials than on congruent trial. Whereas the ERP amplitude of the DD group was larger on congruent trials than on incongruent trials. This result is different from our research hypothesis. Before the present study, we believed that DD children need to invest more cognitive resources in performing the Stroop test than non-dyslexic children due to the inhibitory deficits.

On the congruent trials, it is in line with our research hypothesis that the brain activation of DD children was higher than non-dyslexic children. However, on the incongruent trials, the brain activation was enhanced in non-dyslexic children and weakened in DD children, and the brain activation of non-dyslexic children was larger than DD children.

DD children need to invest more cognitive resources in reading words and sentences than non-dyslexic children. The resource limitation theory of Kahneman suggests that an individual's cognitive capacity is limited.^[19]^ The Stroop test requires participants to judge the color of the words and inhibit the reading words response. Therefore, cognitive resources of DD children may be overloaded when performing the highly conflicting two-in-one stimulus of incongruent trials, resulting in weakened brain activation when judging colors.

P2 is an ERP component of task-related target discrimination, which is related to consciousness activities and reflects the initial semantic processing and attention distribution.^[20]^ The amplitude of P2 of the DD group of congruent trials was significantly higher than incongruent trials, while the amplitude of P2 of the non-dyslexic group of incongruent trials was significantly higher than congruent trials.

**Figure 4:**
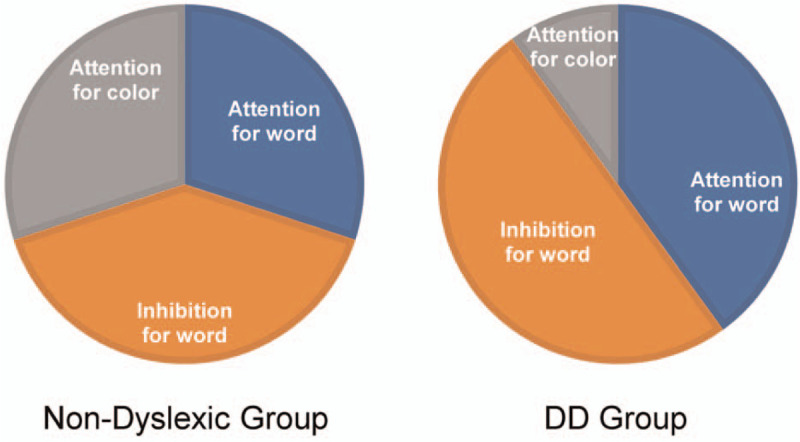
The pattern of cognitive resources allocation between DD children and non-dyslexic children: Stroop test as an example. DD = developmental dyslexia.

P2 was the focus of this study for the following 2 reasons. Most previous studies have found that the Stroop interference occurs after the stimulus-encoding stage.^[21]^ In other words, the interference arises during response-production. P2 indexes some form of selective attention. We found a significant difference in the P2 amplitude of congruent trials and incongruent trials of DD children and non-dyslexic children, indicating differences in the allocation of attentional resources between DD children and non-dyslexic children in the primary stage. It shows that the Stroop interference may not only occur in response-production stage, but also in stimulus-encoding stage. The significant difference in P2 between DD children and non-dyslexic children is a core finding of the present study. We hypothesize that DD children may go through such a cognitive process when performing incongruent trials of the Stroop test: due to reading difficulties, DD children need to invest a lot of cognitive resources in word recognition and naming, while inhibiting word recognition and naming requires more cognitive resources, which leads to insufficient allocation of attention resources in judging colors, and the amplitude of P2 of DD children is wakened on incongruent trials. DD children may be distracted by other stimuli while reading, but non-dyslexic children do not need to invest too many cognitive resources while reading, so they can inhibit interference stimuli (our hypothesis on the distribution pattern of cognitive resources of DD children and non-dyslexic children is shown in Fig. 4).

P3b component reflects the top-down attention distribution, and P3b is related to attention, working memory, and advanced cognitive functions.^[22]^ In the DD group, the P3b amplitude was larger on congruent trials than on incongruent trials, which we think is similar to the results of P2, reflecting inhibition and attention deficit of DD children. In the non-dyslexic group, the P3b latencies were longer on incongruent trials than on congruent trials. We think there may be 2 reasons for this: incongruent trials were more difficult and participants needed more time to judge the color. Affected by the amplitude of P2. The non-dyslexic group had a larger P2 amplitude on incongruent trials than on congruent trials, which prolong the latency of P3b on incongruent trials. According to a previous study on Stroop,^[23]^ researchers believed that if the latencies of P3b is significantly different in response to congruent and incongruent stimuli, indicating that Stroop interference occurs before the response-production. This is the second evidence from the present study that Stroop interference may occur not only in the selection response stage, but also in the stimulus-encoding stage.

In the behavioral data, we found the RT of the DD group was longer than the non-dyslexic group, and the accuracy of the DD group was lower than the non-dyslexic group. The Stroop effect of DD children is stronger, which is consistent with previous studies.^[24,25]^ For DD children, it takes more time to read the word, resulting in a longer latency for color naming and thus a larger interference effects,^[26]^ suggesting poor inhibitory abilities of DD children. Our study found differences between DD and non-dyslexic children in early attentional components such as P2, suggesting the prolonged color naming of the incongruent trials by DD children may be due to resource input of early attentional processes.

What is known is that the more advanced the central processing unit, the more efficient it is. And this may be similar to the brains of DD children and non-dyslexic children when reading. Children with dyslexia read less efficiently than non-dyslexic children under conditions that consume the same cognitive resources. A growing number of studies have revealed that children with dyslexia have magnocellular defect and abnormalities in the cerebral cortex.^[27]^ In the following research, we can use functional magnetic resonance imaging and positron emission tomography to explore the defects in specific brain regions of children with dyslexia.

There are several limitations of the present study. First, we have not explored the inhibitory ability of DD children longitudinally from the neurophysiological aspect. In the following research, we could perform an intervention and prospectively follow up. Second, although the Stroop test is considered to be a representative paradigm for measuring inhibition ability, it cannot separate the interference of textual factors on children with dyslexia. In the next study, it can be combined with flanker and Go/No-go task for further verification. Third, relatively small sample size may limit the deduction from the results. Given these limitations, what we have found in this study should be interpreted prudently. Furthering studies in larger sample sizes and children with dyslexia in different languages are needed to confirm the observations of this study.

## 5 Conclusion

In this study, we explored the Stroop interference effect in children with dyslexia and non-dyslexic children. We found significant differences in behavioral and ERP responses between DD and non-dyslexic children. DD children had larger brain activation on congruent trials than on incongruent trials, whereas non-dyslexic children had larger brain activation on incongruent trials than on congruent trials. The opposite activation patterns may be related to poor inhibition ability of DD children, and this poor inhibition ability may arise from excessive resource input during attention.

## Acknowledgments

The authors thank all children who participated in this study.

## Author contributions

**Investigation:** Yan Luo.

**Methodology:** Qi Jiang, Yan Luo, Ji Long, Xiujuan Tai, Shuqing Liu.

**Resources:** Yan Luo.

**Supervision:** Yan Luo, Shuqing Liu.

**Writing – original draft:** Chengwei Shen.

**Writing – review & editing:** Chengwei Shen, Qi Jiang, Xiujuan Tai, Ji Long, Yan Luo.
